# Identification of Potential Prognostic Biomarker for Predicting Survival in Multiple Myeloma Using Bioinformatics Analysis and Experiments

**DOI:** 10.3389/fgene.2021.722132

**Published:** 2021-09-10

**Authors:** Jian Zhou, Menghui Zhang, Yan Zhang, Xi Shi, Linlin Liu, Ruosi Yao

**Affiliations:** ^1^Department of Hematology, The Affiliated Hospital of Xuzhou Medical University, Xuzhou, China; ^2^Artificial Auditory Laboratory of Jiangsu Province, Xuzhou, China; ^3^College of Medical Imaging, Xuzhou Medical University, Xuzhou, China; ^4^Xuzhou Ruihu Health Management and Consulting Co., Ltd., Xuzhou, China

**Keywords:** multiple myeloma, bioinformatics, RRM2, osalmid, cell cycle

## Abstract

Multiple myeloma (MM) is a malignant disease of plasma cells, which remains incurable because of its unclear mechanism and drug resistance. Herein, we aimed to explore new biomarkers and therapeutic targets in MM. After screening differentially expressed genes (DEGs) in GSE6477 and GSE13591 dataset, we performed Gene Ontology and Kyoto Encyclopedia of Genes and Genomes pathway enrichment analyses of DEGs using DAVID online database. The results indicated that the downregulated DEGs were mainly enriched in the immune-associated biological process. The protein–protein interaction network was constructed by STRING database, on which we performed module analysis and identified key genes. Gene set enrichment analysis (GSEA) and Kaplan–Meier analysis showed that RRM2 could be a novel biomarker in MM diagnosis. We further confirmed that novel RRM2 inhibitor osalmid inhibited MM cell proliferation and triggered cell cycle S phase arrest. Targeting RRM2 was expected to develop new therapeutic strategies for malignant MM.

## Background

Multiple myeloma (MM) is an incurable plasma cell malignancy, accounting for approximately 10% of all hematological diseases (Anderson et al., 2011). Over the past few decades, the treatment options for MM are continuing to improve, leading to significantly prolonged survival. Proteasome inhibitors (bortezomib, carfilzomib, and Ixazomib), immunomodulatory drugs (thalidomide and lenalidomide), and monoclonal antibody drugs (daratumumab) have greatly improved the survival rate for MM patients, but it remains incurable and usually occurs in highly relapsed rate (Demo et al., 2007; Kumar et al., 2008; Chauhan et al., 2011). Despite great advancement in antimyeloma therapy, the acquisition of drug resistance is a great limitation of myeloma treatment. Better understanding of drug resistance and MM progression mechanisms will contribute to the development of potential treatment. Therefore, it’s important to explore new prognosis biomarkers for screening and diagnosis of MM.

Following the improvement of microarray and RNA-sequencing technology, many gene expression studies about MM have been reported in the past few years. In 2016, the Gene Expression Omnibus (GEO) database is built and maintained by the National Center for Biotechnology Information (NCBI) (Clough and Barrett, 2016), which collects lots of high-throughput microarray, chips submitted by researchers from all over the world. The differentially expressed genes (DEGs) between MM patients and healthy donors are screened using the GEO database; then, the biological process and gene regulatory networks are further analyzed (Zhou et al., 2015; Yan et al., 2019). Eventually, to verify the clinical significance of these critical genes, the overall survival (OS) correlation between critical genes and MM patients is explored in the website of Kaplan–Meier plotter.

In the present study, an integrated bioinformatics analysis was performed to pursue the DEGs in MM patients compared with healthy adult donors. First, we downloaded two messenger RNA (mRNA) gene expression profiles [GSE6477 (Chng et al., 2007) and GSE13591 (Agnelli et al., 2009)] from the GEO database and identified the common DEGs from the two microarrays. We further performed the gene ontology (GO) function, KEGG pathway enrichment, and protein–protein interactions (PPI) network analysis. Furthermore, Kaplan–Meier survival analysis demonstrated that GAPDH, RRM2, and TXN were closely associated with MM survival. Meanwhile, biological experiments also demonstrated that RRM2 inhibitor osalmid showed well antitumor activity. Taken together, we identified RRM2 as a biomarker associated with overall survival of patients with MM, and RRM2 inhibitor osalmid could be a possible therapeutic strategy for relapsed and refractory MM.

## Results

### Identification of DEGs in Two GEO Datasets

**FIGURE 1:**
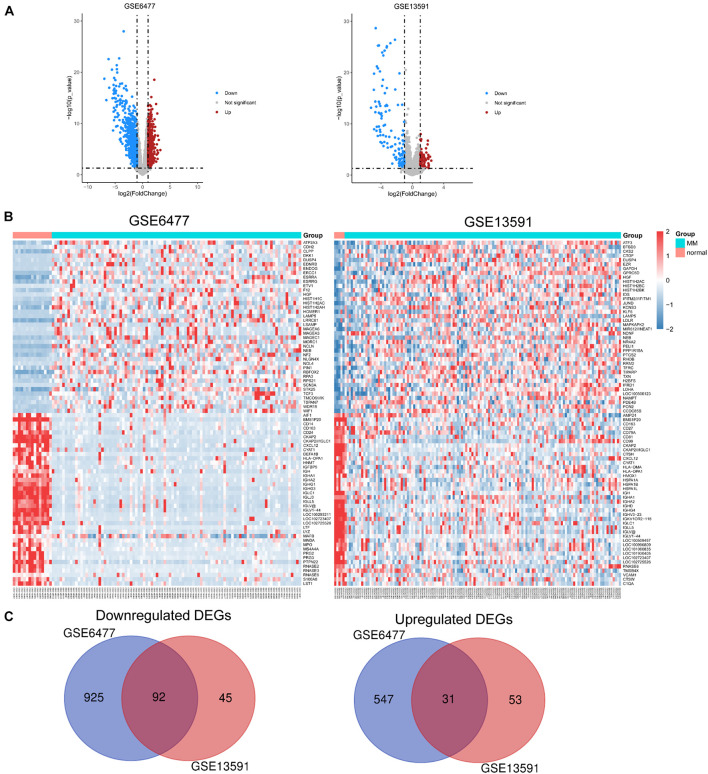
Identification of DEGs in each GEO dataset. **(A)** Volcano plots of the distribution of DEGs in GSE6477 and GSE13591. **(B)** Heatmap of the top 40 DEGs in GSE6477 and GSE13591. **(C)** Commonly downregulated or upregulated DEGs in GSE6477 and GSE13591.

We used expression data of 229 MM patients and 20 healthy adults donors from GSE6477 and GSE13591 datasets. First, we used the robust multiarray standard (RMA) method to perform background correction, quantile normalization, and log transition for raw data (Supplementary Figure 1). As shown in Figure 1A, volcano plots were used to show the distribution of differential expressed genes between MM patients and healthy donors from GSE6477 to GSE13591. Red dots represent significantly upregulated genes (*p* < 0.05, —log_2_FC— > 1), and blue dots represent downregulated genes (*p* < 0.05, —log_2_FC— < −1). Heat maps showed the top 40 differential expressed genes in these two GEO datasets (Figure 1B). Each column denoted a MM or donor sample, and each row represented a significant expressed gene. We totally identified 1,017 downregulated genes and 578 upregulated genes from GSE6477 dataset. Again, we obtained 137 downregulated genes and 84 upregulated genes from GSE13591 dataset. Among them, we obtained common DEGs from the 2 datasets including 92 downregulated and 31 upregulated genes (Figure 1C).

### GO Functional Analysis of DEGs

**FIGURE 2:**
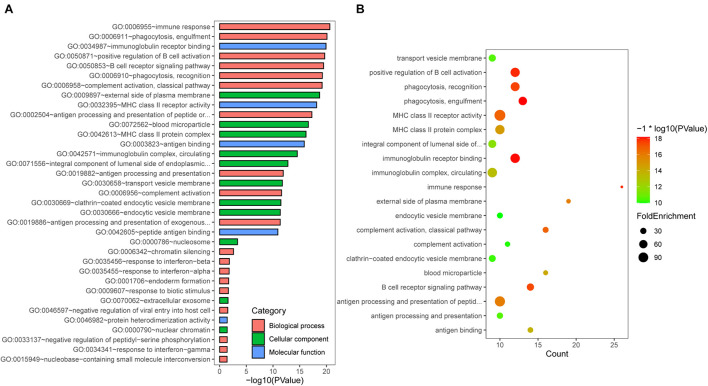
Gene Ontology (GO) enrichment analysis. **(A)** GO analysis of DEGs consisting of biological process (BP), molecular function (MF), and cellular component (CC). **(B)** The bubble map of pathway enrichment analysis of all DEGs.

To further explore the biological function of DEGs identified above, we used GO enrichment analysis using DAVID bioinformatics resources (Huang et al., 2009). The up- or downregulated genes-associated functional categories are shown in Table 1. As shown in Figure 2A, the top 5 biological processes (BPs) contains “immune response,” “phagocytosis, engulfment,” “positive regulation of B cell activation,” “B cell receptor signaling pathway,” and “phagocytosis, recognition.” The top 5 molecule functions (MFs) includes “immunoglobulin receptor binding,” “MHC class II receptor activity,” “antigen binding,” “peptide antigen binding,” and “protein heterodimerization activity.” In the cellular components (CCs) group, the top 5 CCs contains “external side of plasma membrane,” “blood microparticle,” “MHC class II protein complex,” “immunoglobulin complex, circulating,” and “transport vesicle membrane.” Additionally, the bubble map of the entire DEGs was drawn using the ggplot2.R package for the significant biological processes based on *p-*value, and the results indicated that the DEGs were closely associated with immune-related biological processes, such as “positive regulation of B cell activation,” “MHC class II receptor activity,” “immunoglobulin receptor binding,” and “immunoglobulin complex, circulating” (Figure 2B).

**TABLE 1:** Gene Ontology term enrichment analysis of DEGs in multiple myeloma.

**Term**	**Description**	**Count**	***P*-value**
**Upregulated**			
GO:0000786	Nucleosome	4	4.41E-04
GO:0006342	Chromatin silencing	3	2.54E-03
GO:0035456	Response to interferon-beta	2	1.49E-02
GO:0035455	Response to interferon-alpha	2	1.66E-02
GO:0001706	Endoderm formation	2	1.98E-02
GO:0009607	Response to biotic stimulus	2	2.31E-02
GO:0070062	Extracellular exosome	10	2.63E-02
GO:0046597	Negative regulation of viral entry into host cell	2	2.96E-02
GO:0046982	Protein heterodimerization activity	4	3.72E-02
GO:0000790	Nuclear chromatin	3	3.76E-02
**Downregulated**			
GO:0006955	Immune response	25	2.31E-21
GO:0006911	Phagocytosis, engulfment	13	7.84E-21
GO:0034987	Immunoglobulin receptor binding	12	1.19E-20
GO:0050871	Positive regulation of B cell activation	12	2.12E-20
GO:0050853	B cell receptor signaling pathway	14	3.39E-20
GO:0006910	Phagocytosis, recognition	12	5.86E-20
GO:0006958	Complement activation, classical pathway	16	6.52E-20
GO:0009897	External side of plasma membrane	19	1.85E-19
GO:0032395	MHC class II receptor activity	10	6.85E-19
GO:0002504	Antigen processing and presentation of peptide or polysaccharide antigen via MHC class II	10	5.26E-18

### Signaling Pathway Enrichment Analysis of DEGs

**FIGURE 3:**
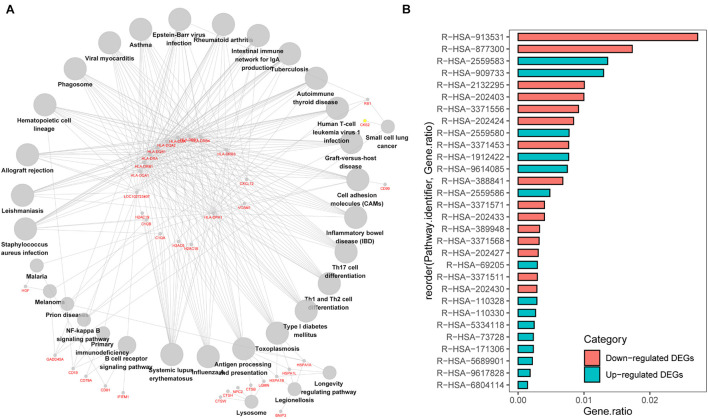
KEGG pathway enrichment analysis of DEGs. **(A)** The correlation between DEGs and KEGG processes. **(B)** The Reactome pathway enrichment analyses of downregulated or upregulated DEGs.

We first analyzed the correlation between DEGs and KEGG processes and found that they were significantly enriched in “systemic lupus erythematosus,” “influenza A,” “antigen processing and presentation,” “Th cell differentiation,” “hematopoietic cell lineage,” “intestinal immune network for IgA production,” and “graft-vs.-host disease” (Figure 3A). Then, we conducted the Reactome pathway enrichment analyses, and the results showed that the enrichment of downregulated DEGs were mainly in the immune system, adaptive immune system, antigen processing and presentation, major histocompatibility complex (MHC) class II antigen presentation, translocation of ZAP-70 to immunological synapse, phosphorylation of CD3 and T-cell receptor (TCR) zeta chains and graft-vs.-host disease, while upregulated DEGs only contained a small amount of genes and were mostly enriched in senescence-associated biological events (Figure 3B and Table 2). Taken together, the downregulated DEGs from the GSE6477 to GSE13591 GEO datasets play an important role in MM progression.

**TABLE 2:** Signaling pathway enrichment analysis of DEGs in multiple myeloma.

**Pathway**	**Name**	**Gene count**	***P*-value**	**Genes**
**Upregulated DEGs**				
Reactome:R-HSA-2559586	DNA damage/telomere stress induced senescence	4	9.01E-04	HIST2H2AA3;HIST2H2AA4; HIST1H2AC;HIST1H1C
Reactome:R-HSA-6804114	TP53 regulates transcription of genes involved in G2 cell cycle arrest	1	1.46E-03	GADD45A
Reactome:R-HSA-909733	Interferon alpha/beta signaling	2	1.62E-03	IFITM1;IFITM2
Reactome:R-HSA-2559583	Cellular senescence	5	1.92E-03	HIST2H2AA3;HIST2H2AA4;TXN;HIST1H2AC; HIST1H1C
Reactome:R-HSA-9617828	FOXO-mediated transcription of cell cycle genes	1	2.38E-03	GADD45A
Reactome:R-HSA-9614085	FOXO-mediated transcription	2	3.12E-03	GADD45A;TXN
Reactome:R-HSA-5689901	Metalloprotease DUBs	3	3.32E-03	HIST2H2AA3;HIST2H2AA4;HIST1H2AC
Reactome:R-HSA-1912422	Pre-NOTCH expression and processing	4	3.36E-03	HIST2H2AA3;HIST2H2AA4;ST3GAL6; HIST1H2AC
Reactome:R-HSA-2559580	Oxidative stress induced senescence	4	3.45E-03	HIST2H2AA3;HIST2H2AA4;TXN;HIST1H2AC
KEGG Pathway: hsa05322	Systemic lupus erythematosus	3	3.78E-02	HIST2H2AA3, HIST2H2AA4, HIST1H2AC
**Downregulated DEGs**				
KEGG Pathway: hsa04612	Antigen processing and presentation	15	1.33E-19	HLA-DRB4, HSPA1L, HLA-DMA, HLA-DPB1, HLA-DRA;…
Reactome:R-HSA-202430	Translocation of ZAP-70 to immunological synapse	10	1.11E-16	HLA-DRB4;HLA-DPB1;HLA-DRA;PTPN22;HLA-DRB3;…
Reactome:R-HSA-2132295	MHC class II antigen presentation	13	1.11E-16	HLA-DRB4;HLA-DMA;HLA-DPB1;HLA-DRA;CTSH;…
Reactome:R-HSA-202427	Phosphorylation of CD3 and TCR zeta chains	10	1.11E-16	HLA-DRB4;HLA-DPB1;HLA-DRA;PTPN22;HLA-DRB3;…
Reactome:R-HSA-168256	Immune system	45	1.11E-16	C1QB;C1QA;IGHM;CEBPD;CD81; FGL2;IGHV3-23;…
Reactome:R-HSA-3371571	HSF1-dependent transactivation	3	1.11E-16	HSPA1L;HSPA1B;HSPA1A
Reactome:R-HSA-3371453	Regulation of HSF1-mediated heat shock response	3	1.11E-16	HSPA1L;HSPA1B;HSPA1A
Reactome:R-HSA-202403	TCR signaling	9	1.11E-16	HLA-DRB4;HLA-DPB1;HLA-DRA;PTPN22;HLA-DRB3;…
Reactome:R-HSA-1280218	Adaptive immune system	26	8.88E-15	IGHM;CD81;IGHV3-23;PTPN22;CD79A;HLA-DMA;…
KEGG Pathway:hsa05332	Graft-vs.-host disease	10	1.98E-14	HLA-DMA;HLA-DRB4;HLA-DPB1;HLA-DRA;HLA-DRB3;…

### The Immune-Associated Functional Enrichment Analysis of Downregulated DEGs

**FIGURE 4:**
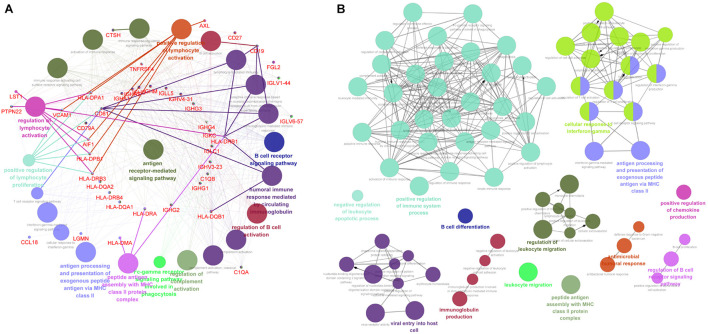
The immune-associated functional enrichment analysis of downregulated DEGs. **(A)** Evaluation of immune system process of downregulated DEGs in ClueGo software. **(B)** Interrelation analysis between pathways in the BPs of downregulated DEGs. Each node represents a GO term, and lines reflect correlations between these terms. The node size is positively associated with term/pathway significance. The node color reflects the functional enrichment groups, with similar function clustering in same color.

The above data indicated that the downregulated DEGs were significantly related with the immune-associated progression. Thus, we analyzed these downregulated DEGs by evaluating the immune system process in ClueGo. As shown in Figure 4A, downregulated DEGs were mainly enriched in antigen processing and presentation of exogenous peptide antigen via MHC class II, B-cell receptor signaling pathway, positive regulation of lymphocyte activation, peptide antigen assembly with MHC class II protein complex, and regulation of lymphocyte activation. Furthermore, we also conducted an interrelation analysis between pathways in the BPs of downregulated DEGs. As expected, we observed an enrichment of the downregulated DEGs largely in antigen processing and presentation of exogenous peptide antigen via MHC class II, peptide antigen assembly with MHC class II protein complex, regulation of B-cell receptor signaling pathway, positive regulation of immune system process, and B-cell differentiation (Figure 4B).

### PPI and GSEA Analysis of Immune-Related Function of Hub Genes

**FIGURE 5:**
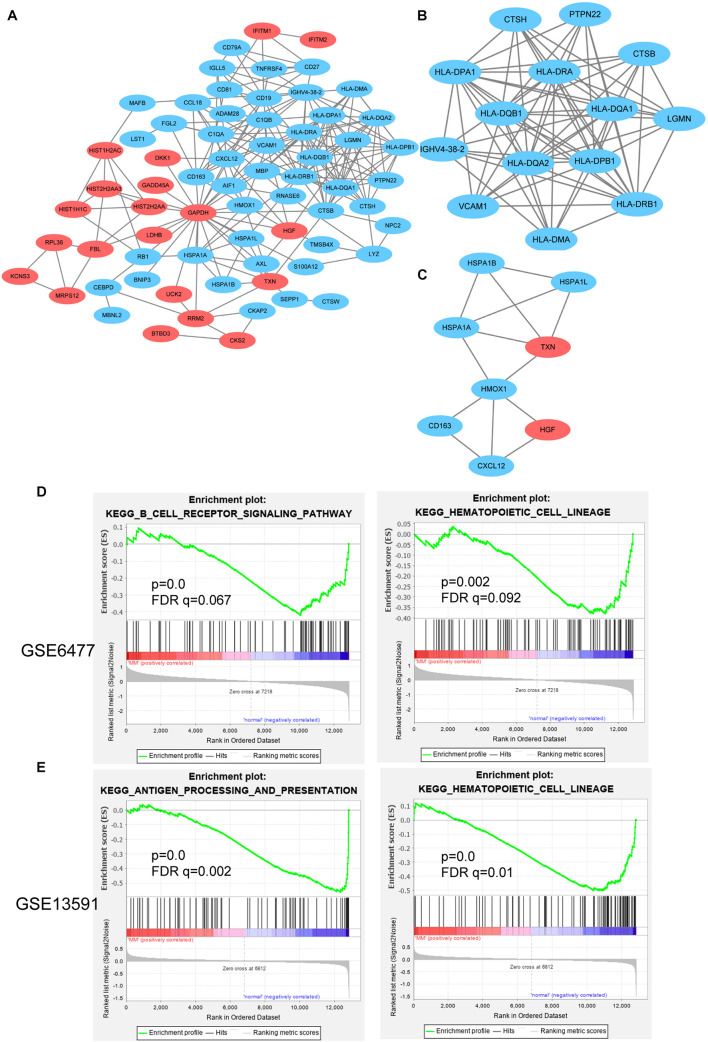
PPI network and GSEA analysis for hub genes. **(A)** PPI network of DEGs contained down- and upregulated genes. Cluster analysis of PPI network in Cytotype MCODE: module 1 **(B)** and module 2 **(C)**. GSEA analysis of immune-related function in GSE6477 **(D)** and GSE13591 **(E)**.

STRING is an online analysis tool, which is often used to study and integrate interaction between proteins (Szklarczyk et al., 2017). Based on the DEGs identified above, we constructed the protein–protein interaction (PPI) network of DEGs expression in MM. After removing the separated nodes, we described a complex PPI network of entire DEGs, and the final PPI network contained 68 nodes and 222 edges (Figure 5A). Subsequently, we utilized cluster analysis of PPI network in Cytotype MCODE; we identified two significant modules: module 1 and module 2. Module 1 contained 14 nodes and 72 edges (Figure 5B); the DEGs in module 1 were mainly enriched in adaptive immune system, immune system, and MHC class II antigen presentation (Table 3). Inversely, module 2 includes 8 nodes and 13 edges (Figure 5C); the DEGs in module 2 were not relevant for immune system (Table 4).

**TABLE 3:** Pathway enrichment analysis of Module 1 genes function.

**Term**	**Description**	**Count**	***P*-value**
R-HSA-202430	Translocation of ZAP-70 to immunological synapse	8	1.11E-16
R-HSA-2132295	MHC class II antigen presentation	11	1.11E-16
R-HSA-202427	Phosphorylation of CD3 and TCR zeta chains	8	1.11E-16
R-HSA-202403	TCR signaling	8	1.11E-16
R-HSA-389948	PD-1 signaling	7	1.11E-16
R-HSA-202433	Generation of second messenger molecules	7	1.11E-16
R-HSA-1280218	Adaptive immune system	13	1.11E-16
R-HSA-877300	Interferon gamma signaling	8	1.11E-16
R-HSA-202424	Downstream TCR signaling	7	1.11E-16
R-HSA-168256	Immune system	13	1.11E-16
R-HSA-913531	Interferon signaling	8	1.11E-16
R-HSA-388841	Costimulation by the CD28 family	7	1.11E-16
R-HSA-1280215	Cytokine signaling in immune system	8	1.11E-16
R-HSA-1679131	Trafficking and processing of endosomal TLR	2	6.86E-04

**TABLE 4:** Pathway enrichment analysis of Module 2 genes function.

**Term**	**Description**	**Count**	***P*-value**
R-HSA-3371568	Attenuation phase	3	1.11E-16
R-HSA-3371571	HSF1-dependent transactivation	3	1.11E-16
R-HSA-3371453	Regulation of HSF1-mediated heat shock response	3	1.11E-16
R-HSA-3371556	Cellular response to heat stress	3	1.11E-16
R-HSA-3371511	HSF1 activation	3	1.11E-16
R-HSA-2262752	Cellular responses to stress	4	1.11E-16
R-HSA-8953897	Cellular responses to external stimuli	4	1.11E-16
R-HSA-9662834	CD163 mediating an anti-inflammatory response	1	3.13E-04
R-HSA-3371497	HSP90 chaperone cycle for steroid hormone receptors (SHR)	3	4.26E-04
R-HSA-6785807	Interleukin-4 and Interleukin-13 signaling	2	6.21E-04
R-HSA-1251985	Nuclear signaling by ERBB4	1	3.39E-03
R-HSA-8875513	MET interacts with TNS proteins	1	9.13E-03
R-HSA-8865999	MET activates PTPN11	1	9.13E-03
R-HSA-8875791	MET activates STAT3	1	9.13E-03

GSEA is a strong analytical method to identify the differential genes enrichment in several biological events (Saxena et al., 2006). Specifically, in GSE6477 cohort, the low expression of CD79A, CD19, and CD81 were in direct proportion to the B-cell receptor signaling pathway. The downregulated HLA-DRA, HLA-DRB4, HLA-DRB1, and CD19 genes were enriched in response to hematopoietic cell lineage (Figure 5D). In another GSE13591 dataset, we found that 12 downregulated genes were positively correlated with antigen processing and presentation, including HLA-DRB4, CTSB, HLA-DQB1, HLA-DRB1, HLA-DRA, HLA-DPB1, LGMN, HSPA1B, HLA-DQA1, HSPA1A, HLA-DMA, and HLA-DPA1 (Figure 5E).

### Identification of Highly Expressed Hub Genes by CytoHubba Methods

**FIGURE 6:**
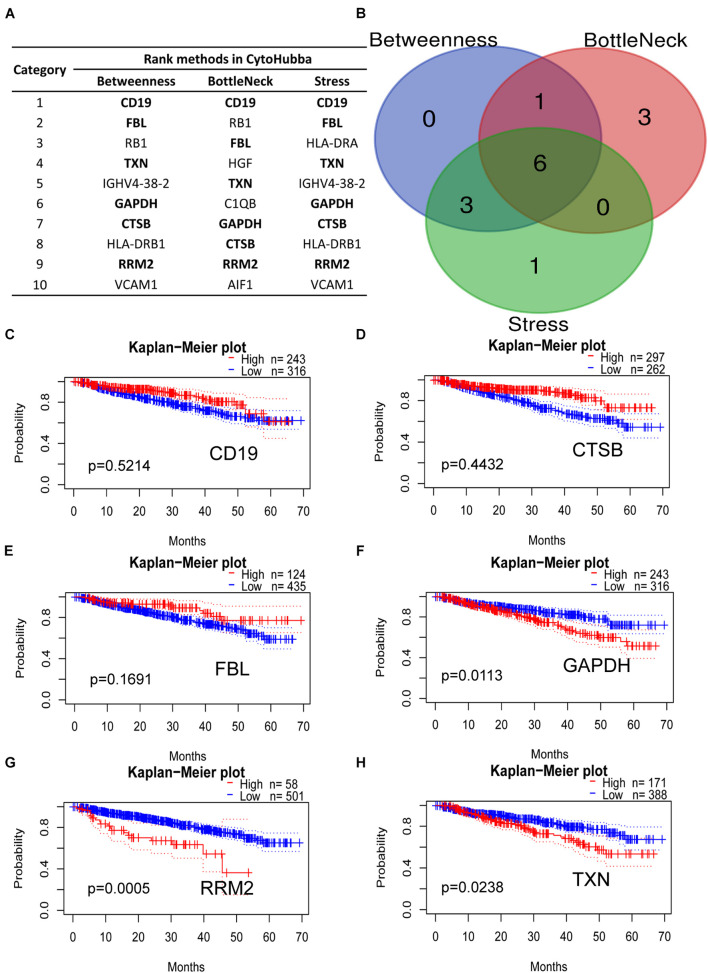
Hub genes ranked by three CytoHubba methods. **(A)** Bold gene symbols were the overlap genes in top 10 by three ranked methods. **(B)** Overlapped six genes in Betweenness, Bottleneck, and Stress methods. **(C–H)** Kaplan–Meier analysis of CD19, CTSB, FBL, GAPDH, RRM2, and TXN correlated with MM progression.

The DEGs mentioned in the PPI network analysis were further scored in top 10 by three methods in CytoHubba, which were selected as key hub genes of MM (Figure 6A). The common genes included CD19, CTSB, FBL, GAPDH, RRM2, and TXN, which may play a vital role in MM progression (Figure 6B). Subsequently, our Kaplan–Meier analysis showed that CD19, CTSB, and FBL were not relevant to the OS of MM patients (Figures 6C–E, *p* > 0.05). On the contrary, GAPDH, RRM2, and TXN were closely associated with MM survival (Figures 6F–H, *p* < 0.05). Among them, the housekeeping gene GAPDH did not participate in MM progression. As a major player in the antioxidant pathway, TXN probably played a critical role in reducing oxidized cysteine residues and cleaving disulfide bonds (Cao et al., 2020; Yang et al., 2020). Surprisingly, it has been reported that silencing RRM2 inhibits multiple myeloma by targeting the Wnt/β−catenin signaling pathway (Liu et al., 2019), implying that RRM2 may be a new therapeutic target for MM. Therefore, we conducted various experiments to evaluate the biological functions by commercial specific RRM2 inhibitor osalmid in MM cell lines.

### RRM2 Inhibitor Osalmid Showed Antitumor Activity

**FIGURE 7:**
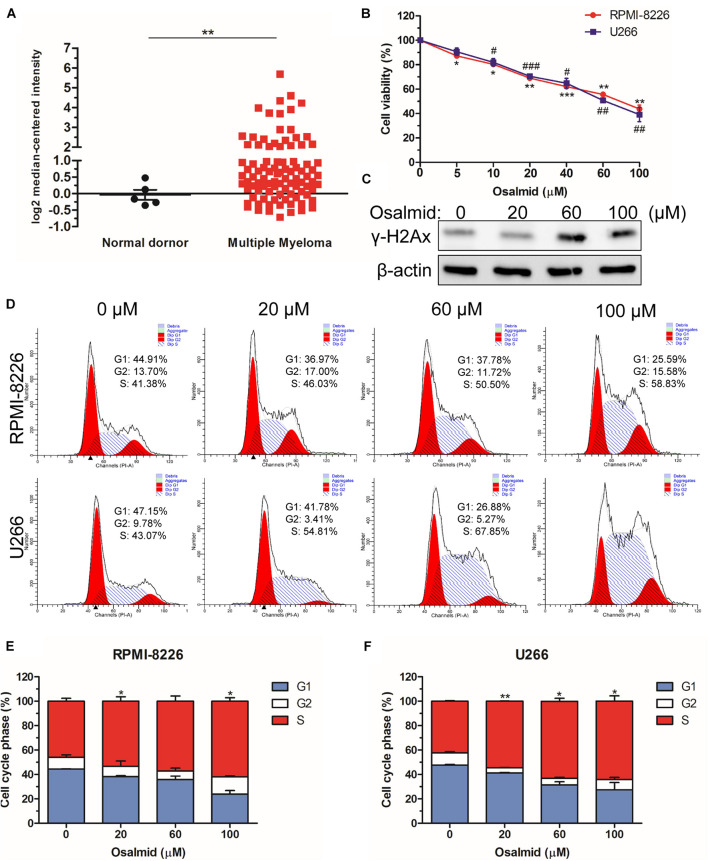
RRM2 inhibitor osalmid showed antitumor activity. **(A)** The mRNA expression level of RRM2 between MM patients and normal donors using the Oncomine database. **(B)** CCK8 assay demonstrated an inhibition of RPMI-8226 and U266 cell proliferation following osalmid treatment. **(C)** Western blot assay of γH2Ax protein expression in U266 cells treated with different doses of osalmid. **(D)** Flow cytometry assay analyzed RPMI-8226 and U266 cell cycle distribution treated by osalmid. **(E,F)** Statistical analysis of cell accumulation of cells in S phase in RPMI-8226 and U266 cells following treatment with osalmid. Error bars indicate mean ± SD. ^#,^*p < 0.05, ^##,^**p < 0.01, ^###,^***p < 0.001.

We further evaluated the mRNA expression level of RRM2 between MM patients and normal donors using the Oncomine database; the results showed a significant elevation of mRNA level of RRM2 in MM patients (Figure 7A). As a novel identified RRM2 inhibitor, osalmid has been approved for treating cholecystitis, inflammation, and postcholecystectomy syndrome, which inhibited RRM2 activity by binding to hydrogen bond of RRM2 (Liu et al., 2016). Herein, we used CCK8 cell viability assay to explore the effect of osalmid on MM cells; the results showed that osalmid inhibited RPMI-8226 and U266 cell viabilities in a concentration-dependent manner (Figure 7B). RRM2 is a member of ribonucleotide reductases, which is required for DNA synthesis. Thus, we further evaluated the impact of osalmid on DNA damage. Our data confirmed that RRM2 inhibitor osalmid could activate the expression of γH2Ax, which is a hallmark of DNA damage (Figure 7C). Subsequently, flow cytometry assay indicated that RRM2 inhibitor osalmid induced MM cell cycle S phase arrest in a dose-dependent manner (Figures 7D–F). Although osalmid could also inhibit normal cells, it was not relevant to the OS of acute myeloid leukemia (AML), diffuse large B-cell lymphoma (DLBCL), and follicular lymphoma patients (Supplementary Figures 2, 3). Totally, RRM2 could be an important experimental new agent for treatment of MM.

## Discussion

Multiple myeloma incidence has been increasing over the past few decades, making it a disease of considerable clinical and economic impact (Ferlay et al., 2015). In this study, we screened out DEGs based on GSE6477 and GSE13591 datasets. The upregulated DEGs were strikingly enriched in senescence-associated biological events, and the downregulated DEGs in immune system, adaptive immune system, antigen processing and presentation, MHC class II antigen presentation, translocation of ZAP-70 to immunological synapse, phosphorylation of CD3 and TCR zeta chains, and graft-vs.-host disease. It is well known that immunomodulation plays an important role in MM progression, implying that the downregulated DEGs could be new targets in developing novel immunomodulatory drugs.

In recent years, immunomodulatory drugs (IMiDs) are broadly applied in the treatment of multiple myeloma. At present, three IMiDs, namely, thalidomide, lenalidomide, and pomalidomide, have been approved for the treatment of MM (Singhal et al., 1999). Cereblon (CRBN), a common target for IMiDs, was part of E3 ubiquitin ligase complex. IMiD drugs bound to CRBN and triggered a change in CRBN targets, ultimately initiating therapeutic activity (Zhu et al., 2011; Chamberlain et al., 2014). The resistant mechanism to IMiDs was due to downregulation of CRBN expression or CRBN mutations (Kortum et al., 2016). In our study, we identified several hub genes using gene set enrichment analysis. Meanwhile, we conducted Kaplan–Meier analysis; lower expression levels of HLA-DPB1, HSPA1A, HSPA1B, and TNFRSF4 showed worse overall survival (OS) in MM patients.

Bioinformatics is a data-driven branch of science that has been commonly used for high-through data analysis and involves a large number of powerful analysis software packages and database (Xia, 2017). We further identified top 10 genes using Betweenness, Bottleneck, and Stress methods in Cytohubba. Kaplan–Meier analysis indicated that GAPDH, RRM2, and TXN were closely associated with MM survival (*p* < 0.05). Among them, RRM2 has a crucial role in mediating cancer cell survival and progression (Liu et al., 2016; Tang et al., 2020). Therefore, we used a new identified RRM2 inhibitor osalmid to silence RRM2 expression and evaluate its role in MM progression. As expected, silencing RRM2 by osalmid treatment led to cell viability inhibition and cell cycle arrest. Taken together, targeting RRM2 may provide a novel therapeutic strategy for the treatment of multiple myeloma.

## Conclusion

In summary, integrated bioinformatics analysis of datasets of MM patients and healthy adult donors was performed. We identified several genes associated with immune-related biological processes, such as B-cell receptor signaling pathway, cytokine-cytokine receptor interaction, hematopoietic cell lineage, and antigen processing and presentation. Among them, GAPDH, RRM2, and TXN genes were all upregulated, and Kaplan–Meier analysis showed that these genes were closely associated with worse overall survival (OS) of MM. Gene enrichment analysis and biological experiments indicated that RRM2 could be a potential biomarker in MM progression.

## Materials and Methods

### GEO Datasets Selection and Data Processing

GSE6477 and GSE13591 were obtained from GEO^1^ database, which were measured in the GPL96 platform (Affymetrix Human Genome U133A Array). We further analyzed the DEGs using online GEO2R software, and *p* < 0.05 and —log_2_FC— > 1 were used as cutoff criteria.

### GO and KEGG Pathway Enrichment Analysis of DEGs

GO term was used to analyze the biological events including biological process, cellular component, and molecular function by DAVID online biological information database (Huang et al., 2007). Signaling pathway analysis was carried out using another online database, REACTOME. *p* < 0.05 was considered as statistically significant and to achieve significant enrichment.

### PPI Network Analysis of DEGs

The STRING database contained known and predicated protein–protein association data for many organisms, including *Homo sapiens* (Szklarczyk et al., 2017). The Cytoscape software V3.5.1 was used to show the PPI network and the most significant module based on the MCODE score and node number (Shannon et al., 2003).

### Gene Set Enrichment Analysis

GSEA is a computational method to evaluate whether a set of genes show the significant difference in various biological events (Subramanian et al., 2007). The analysis was conducted with default settings. *p* < 0.05 and false discovery rate (FDR) *q* < 0.25 were considered as statistically significant.

### Cell Viability Assay

Indicated cells were plated at 1 × 10^4^ cells per 96-well and treated with different concentrations of osalmid. The cell survival rate was detected using CCK8 kit according to the protocol (KeyGEN, Nanjing, China).

### Western Blot

Indicated cells with different dose of osalmid were lysed in the radioimmunoprecipitation assay (RIPA) buffer, and the detailed procedure was conducted according to the previous report (Yao et al., 2017). The following antibodies were used: β-actin and γH2Ax (Proteintech, Wuhan, China).

### Cell Cycle Analysis

The distribution of indicated cells were analyzed by flow cytometry. The experiments were performed according to the procedure described previously (Yao et al., 2018).

### Statistical Analysis

The results are presented as mean ± SD of three independent experiments. The statistical significance was assessed by Student’s *t*-test with #, ^∗^*p* < 0.05 and ##, ^∗∗^*p* < 0.01.

## Data Availability Statement

The original contributions presented in the study are included in the article/Supplementary Material, further inquiries can be directed to the corresponding author/s.

## Author Contributions

JZ and RY conceived and designed the experiments, and analyzed the data. MZ downloaded the GEO datasets and performed DEGs. YZ and XS conducted the GSEA and survival analysis. RY and LL wrote the manuscript. All authors contributed to the article and approved the submitted version.

## Conflict of Interest

RY was employed by company Xuzhou Ruihu Health Management and Consulting Co., Ltd., China. The remaining authors declare that the research was conducted in the absence of any commercial or financial relationships that could be construed as a potential conflict of interest.

## Publisher’s Note

All claims expressed in this article are solely those of the authors and do not necessarily represent those of their affiliated organizations, or those of the publisher, the editors and the reviewers. Any product that may be evaluated in this article, or claim that may be made by its manufacturer, is not guaranteed or endorsed by the publisher.

## Footnotes

1
https://www.ncbi.nlm.nih.gov/geo/

## Abbreviations

MM: multiple myeloma
GEO: gene expression omnibus
DEGs: differentially expressed genes
GO: gene ontology
BPs: biological processes
MFs: molecule functions
CCs: cellular components
KEGG: Kyoto Encyclopedia of Genes and Genomes
PPI: protein–protein interaction
STRING: Search Tool for the Retrieval of Interacting Genes
MCODE: Molecular Complex Detection
GSEA: gene set enrichment analysis
IMiDs: immunomodulatory drugs
RRM2: ribonucleotide reductase M2
OS: overall survival.
