# Supplementary figures and images for: Identification of Potential Prognostic Biomarker for Predicting Survival in Multiple Myeloma Using Bioinformatics Analysis and Experiments
Source: Front Genet. 2021 Sep 10;12:722132. doi: 10.3389/fgene.2021.722132 (PMC8461066; doi:10.3389/fgene.2021.722132)

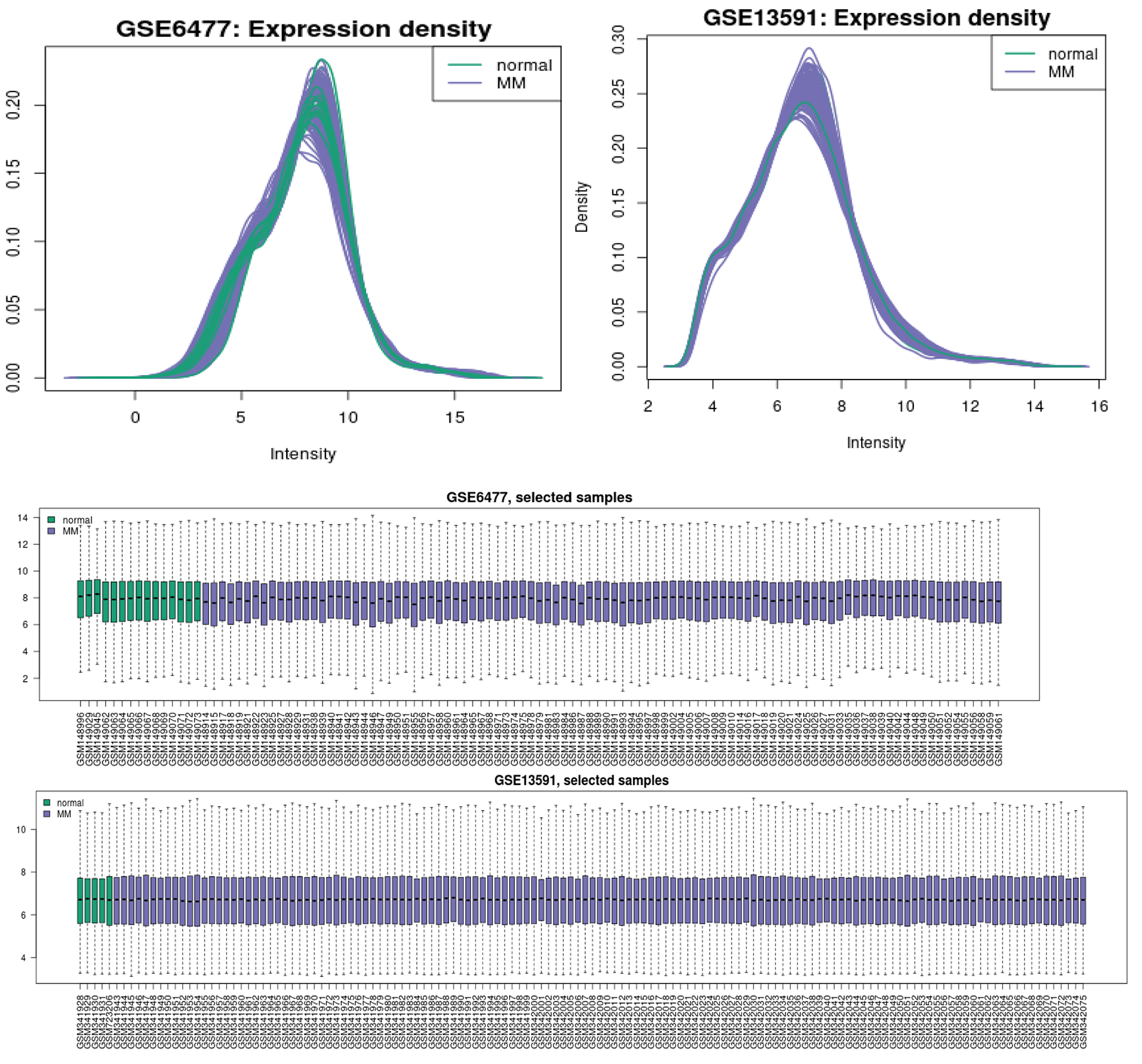

Supplement: Supplementary file 1 [file Image_1.TIF]

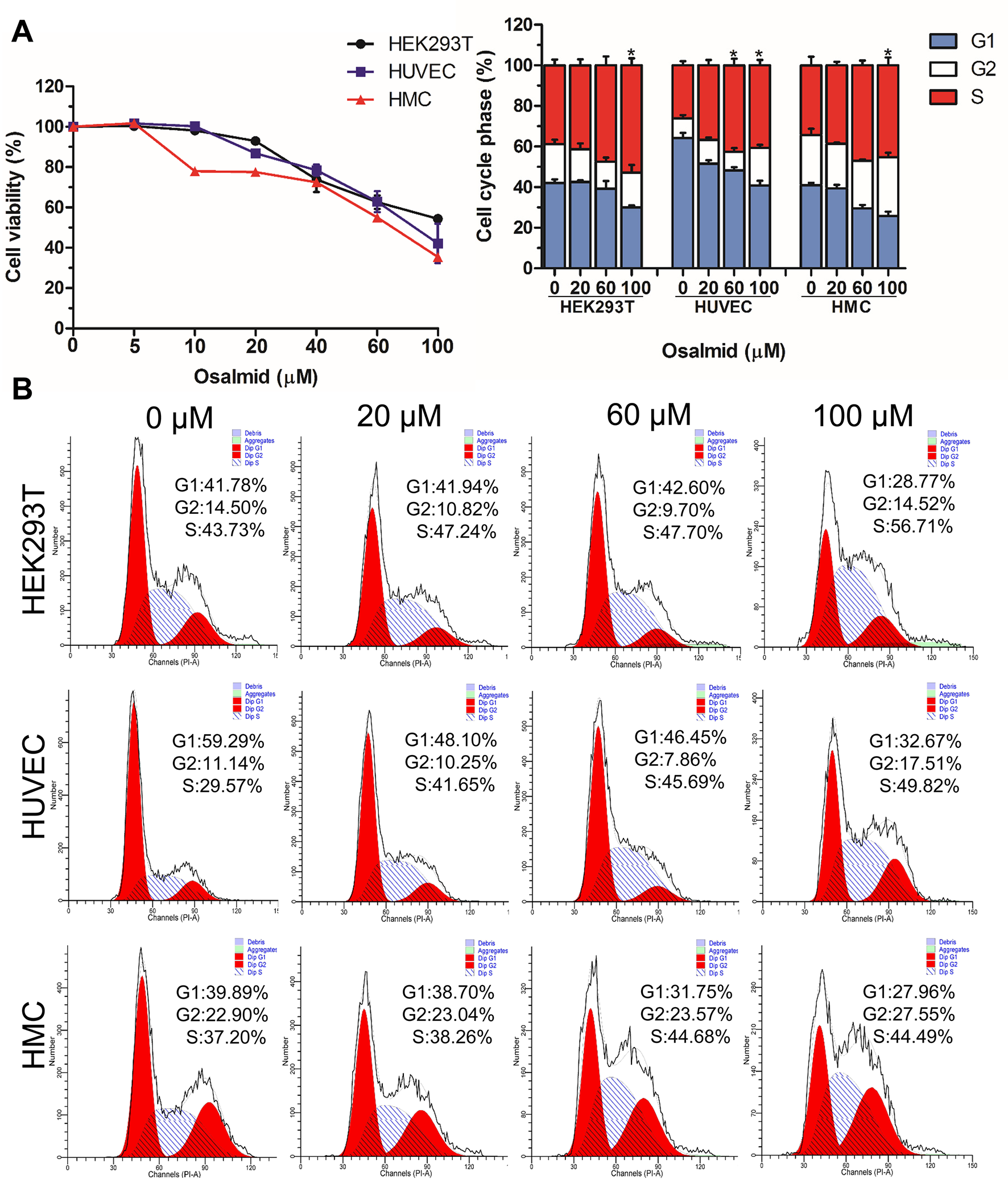

Supplement: Supplementary file 2 [file Image_2.TIF]

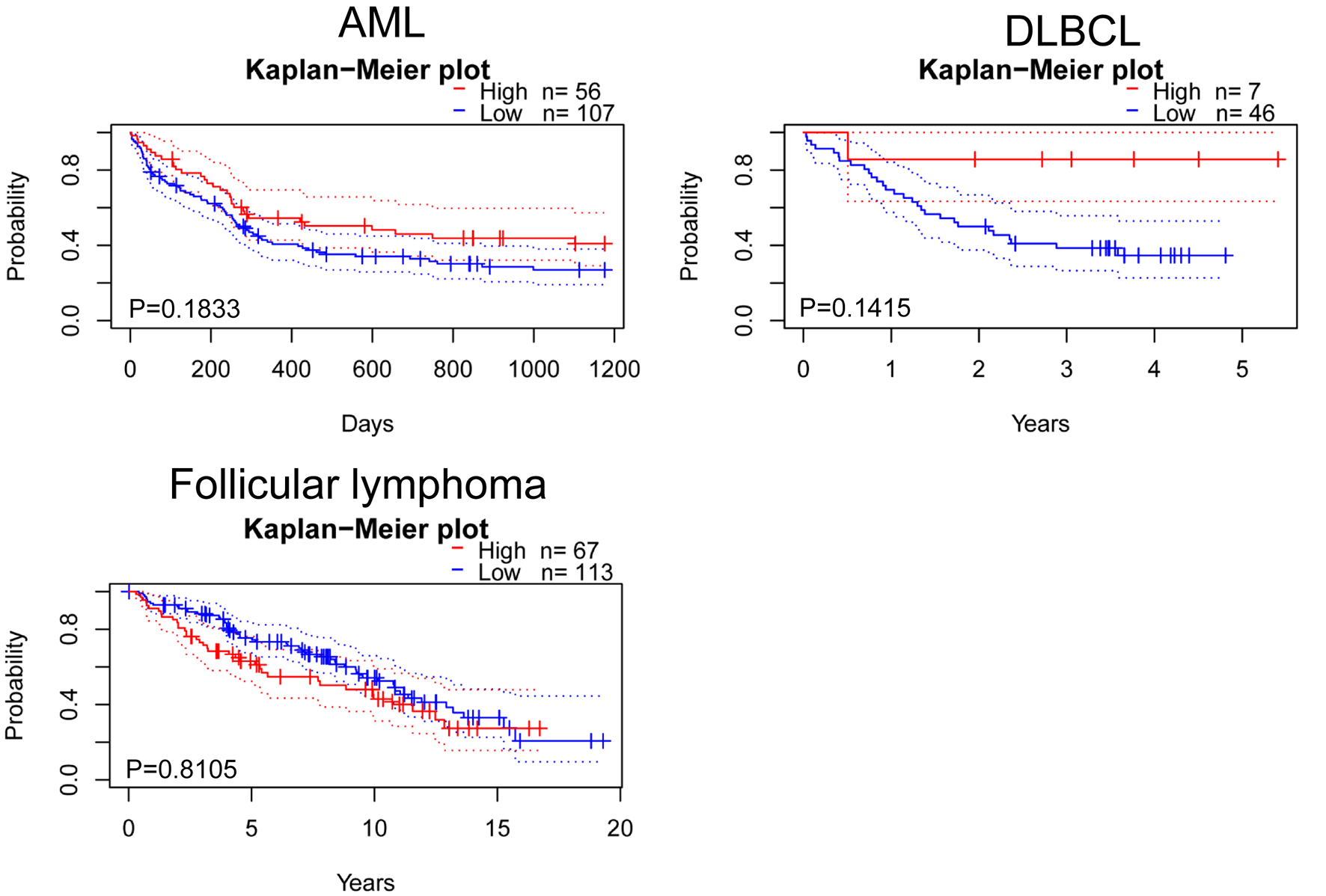

Supplement: Supplementary file 3 [file Image_3.TIF]
