# Supplementary material for: Identification of Potential Prognostic Biomarker for Predicting Survival in Multiple Myeloma Using Bioinformatics Analysis and Experiments
Source: Front Genet. 2021 Sep 10;12:722132. doi: 10.3389/fgene.2021.722132 (PMC8461066; doi:10.3389/fgene.2021.722132)
Supplement: Supplementary file 4 [file Data_Sheet_1.docx]

**Figure S1. The normalization and gene expression distribution of raw data.** Density plots of MM patients and normal donors in the GSE6477 and GSE13591. Boxplots of MM patients and normal donors in the GSE6477 and GSE13591.

**Figure S2. The effect of osalmid on normal cells.** A.CCK8 assay demonstrated an inhibition of HEK293T, HUVEC and HMC cell viabilities following osalmid treatment. B. Flow cytometry assay analyzed HEK293T, HUVEC and HMC cell cycle distribution treated by osalmid. Error bars indicate mean ± SD. #, *p< 0.05.

**Figure S3. Kaplan–Meier analysis of RRM2 correlated with AML, DLBCL and Follicular lymphoma progression.**
